# Insight and Quality of Life in Schizophrenia: Correlations with Clinical and Sociodemographic Factors

**DOI:** 10.1192/j.eurpsy.2026.12134

**Published:** 2026-07-31

**Authors:** Y. Sellaouti, M. Lagha, D. Njah, N. Msadek, Z. Arfaoui, W. Homri

**Affiliations:** 1 Razi Hospital, Manouba, Tunisia; 2 Psychiatry “C”, Razi Hospital, Manouba, Tunisia

## Abstract

**Introduction:**

Schizophrenia is a chronic psychiatric disorder marked by cognitive and emotional disturbances, impaired reality perception, and substantial deterioration in patients’ quality of life. Insight, defined as awareness of the illness and its consequences, is a pivotal determinant of treatment adherence and prognosis. Its influence on quality of life, however, remains controversial, oscillating between improved adherence and heightened psychological distress. Simultaneous assessment of these dimensions may provide a clearer understanding of their interplay and highlight factors shaping their trajectory.

**Objectives:**

To examine the relationship between insight and quality of life, and to identify clinical and sociodemographic factors that may influence these two dimensions in patients with schizophrenia.

**Methods:**

We conducted a cross-sectional study among patients with schizophrenia followed up in the post-cure unit of Psychiatry Department C at Razi Hospital. Insight was assessed using the **Insight Scale Q8 (IS-Q8)**, while quality of life was measured with the **12-Item Short Form Health Survey (SF-12)**, which provides two distinct composite scores: the **Physical Component Summary (PCS)** and the **Mental Component Summary (MCS)**.

**Results:**

A total of 62 patients with schizophrenia were included. The mean age was 43.2 ± 12.7 years, with 79% males (male/female ratio = 4.17). The mean illness duration was 16.7 ± 10.5 years, and the median duration of untreated psychosis (DUP) was 12 [0–42] months. Poor treatment adherence was observed in 68% of patients, and 37% reported psychoactive substance use. The mean **Insight Scale Q8 (IS-Q8)** score was 4.58 ± 3.12, indicating moderate insight, while mean **SF-12** scores were 39/100 for **PCS** and 40/100 for **MCS**, reflecting impaired quality of life.

Better insight was positively correlated with higher quality of life (r = 0.45, p = 0.002). Poor treatment adherence was associated with lower insight (r = 0.50, p < 0.0001) and reduced quality of life (PCS: r = 0.40, p = 0.002; MCS: r = 0.45, p = 0.001). Psychoactive substance use was linked to poorer insight (p = 0.045) and lower physical (p = 0.028) and mental (p = 0.018) health scores. Additionally, a higher number of hospitalizations correlated with lower insight (r = -0.40, p = 0.001) and reduced quality of life (PCS: r = -0.45, p = 0.0002; MCS: r = -0.35, p = 0.005). Longer DUP was also associated with poorer insight (r = -0.35, p = 0.004).

**Conclusions:**

Joint assessment of insight and quality of life is crucial for optimizing schizophrenia management. Better characterization of their determinants may inform more tailored and effective interventions.

**Disclosure of Interest:**

None Declared

